# Exploring alternatives to laparoscopic renal biopsy: a critical examination of safety, efficacy, and costs

**DOI:** 10.1080/0886022X.2024.2343387

**Published:** 2024-04-24

**Authors:** David Fernando Torres Cortes, Daniela Carrascal, Gonzalo Andrés Montaño Rozo, José David Cardona Ortegón, Oscar Mauricio Rivero Rapalino

**Affiliations:** a Department of Diagnostic Imaging, Fundación Santa Fe de Bogotá, Bogotá, Colombia; b Residency in Radiology and Diagnostic Imaging, El Bosque University, Bogotá, Colombia

Dear editor,

We read with great interest the article by Lingling Xu et al. [1] on the safety and efficacy of laparoscopic renal biopsy (LRB). We highly appreciate the authors’ great effort in the methodological development of this study. However, we have a few considerations that we think are relevant to this topic.

This paper has received significant attention due to the increasing global incidence of chronic kidney disease and acute renal injury, the latter which affects 7 out of 100 inpatients and 6 out of 10 critically ill patients. These conditions are associated with a significant burden of comorbidities in affected patients [2]. Consequently, there has been a rise in therapeutic efforts and healthcare costs worldwide. Therefore, many healthcare systems are adopting new diagnostic markers and minimally invasive procedures to support the triple aim of healthcare [3]. Among these procedures, percutaneous renal biopsy (PRB) with ultrasound guidance is the most convenient option for resource optimization, performed by the interventional radiologist with good safety rates [4].

Regarding complications, the well-known factors that increase the risk of bleeding such as arterial hypertension or obesity tend to become more controlled over time; the overall rate of complication in the literature varies widely, ranging from 0.3% to 7.3% for major complications [5]. In our recent institutional experience we have performed 196 renal biopsies from January to October 2023, 140 of them in outpatients and 56 inpatients, 71% were of native kidneys and 29% in transplanted patients, with proteinuria and graft rejection being the most prevalent indications respectively. Of the above a total complication rate of 8.6%, all minor complications (literature search) such as: pain greater than 7/10 on the analogue scale (0.5%), self-resolving hematuria (0.5%) and non-expansive hematoma (7.5%). Five cases (2.5% from total) were transjugular biopsy, the indication for these was always uncorrectable hemorrhagic diathesis, none of these presented complications.

Moreover, when facing special situations such as pediatric patients, morbid obesity, coagulation abnormalities and poor renal architecture, which may make percutaneous renal biopsy (PRB) challenging or contraindicated, the first alternative to consider in a middle-income country is performing a transjugular renal biopsy. This is due to its acceptable accuracy and safety profile, with a technical success rate of 90–100% and a major complication rate of 4% [6,7], also it has many advantages allowing faster recovery time (6–8 h observation) and collection of multiple organ samples [7].

While laparoscopic renal biopsy has been shown to be a safe procedure with a 100% diagnostic accuracy in their cohort [1], patients require at least 24 h of clinical and biomarker follow-up after the procedure, plus drainage during recovery. A cost study performed on transplanted kidneys in 2023 [8] reported costs of around US $2063 in 2019 for a percutaneous renal biopsy, which is a high cost for an outpatient procedure. In our institution, the cost of a PRB is around US $1100 in 2024, a TRB US $761 and for a laparoscopic procedure US $1500; these values do not include other supplies and postoperative medications. This reinforces our thesis in favor of minimally invasive techniques, in the absence of further cost studies.

Finally, we find it valuable to know if you have any consideration when starting comorbidity control in those patients that are at high risk of PRB to decide to perform laparoscopic rather than intravascular procedure.
